# Different sources of omega-3 polyunsaturated fatty acids affects apparent digestibility, tissue deposition, and tissue oxidative stability in growing female rats

**DOI:** 10.1186/1476-511X-10-179

**Published:** 2011-10-14

**Authors:** Janet C Tou, Stephanie N Altman, Joseph C Gigliotti, Vagner A Benedito, Elizabeth L Cordonier

**Affiliations:** 1 Human Nutrition and Foods, Division of Animal and Nutritional Sciences, West Virginia University, P.O. Box 6108, Morgantown, WV 26506 USA; 2 Genetics and Developmental Biology, Division of Plant and Soil Sciences, West Virginia University, P.O. Box 6108, Morgantown, WV 26506 USA; 3 Biochemistry, Division of Animal and Nutritional Sciences, West Virginia University, P.O. Box 6108, Morgantown, WV 26506 USA

**Keywords:** marine oils, flaxseed oil, krill oil, digestibility, tissue accretion, oxidative stress

## Abstract

**Background:**

Numerous health benefits associated with increased omega-3 polyunsaturated fatty acid (n-3 PUFA) consumption has lead to an increasing variety of available n-3 PUFA sources. However, sources differ in the type, amount, and structural form of the n-3 PUFAs. Therefore, the objective of this study was to determine the effect of different sources of ω-3 PUFAs on digestibility, tissue deposition, eicosanoid metabolism, and oxidative stability.

**Methods:**

Female Sprague-Dawley rats (age 28 d) were randomly assigned (n = 10/group) to be fed a high fat 12% (wt) diet consisting of either corn oil (CO) or n-3 PUFA rich flaxseed (FO), krill (KO), menhaden (MO), salmon (SO) or tuna (TO) oil for 8 weeks. Rats were individually housed in metabolic cages to determine fatty acid digestibility. Diet and tissue fatty acid composition was analyzed by gas chromatography and lipid classes using thin layer chromatography. Eicosanoid metabolism was determined by measuring urinary metabolites of 2-series prostaglandins (PGs) and thromoboxanes (TXBs) using enzyme immunoassays. Oxidative stability was assessed by measuring thiobarbituric acid reactive substances (TBARS) and total antioxidant capacity (TAC) using colorimetric assays. Gene expression of antioxidant defense enzymes was determined by real time quantitative polymerase chain reaction (RT-qPCR).

**Results:**

Rats fed KO had significantly lower DHA digestibility and brain DHA incorporation than SO and TO-fed rats. Of the n-3 PUFA sources, rats fed SO and TO had the highest n-3 PUFAs digestibility and in turn, tissue accretion. Higher tissue n-3 LC-PUFAs had no significant effect on 2-series PG and TXB metabolites. Despite higher tissue n-3 LC-PUFA deposition, there was no increase in oxidation susceptibility indicated by no significant increase in TBARS or decrease in TAC and gene expression of antioxidant defense enzymes, in SO or TO-fed rats.

**Conclusions:**

On the basis that the optimal n-3 PUFA sources should provide high digestibility and efficient tissue incorporation with the least tissue lipid peroxidation, TO and SO appeared to be the most beneficial of the n-3 PUFAs sources evaluated in this study.

## Background

The omega-6 polyunsaturated fatty acid (n-6 PUFA), linoleic acid (LA, 18:2n-6) followed by the omega-3 polyunsaturated fatty acid (n-3 PUFA), alpha-linolenic acid (ALA, 18:3n-3) are the primary PUFAs in the Western diet [1]. LA and ALA are essential fatty acids that must be obtained from the diet. Once consumed, LA and ALA may be metabolized in the mammalian tissue into long-chain PUFAs (LC-PUFAs). The major n-6 long-chain polyunsaturated fatty acid (LC-PUFA) is arachidonic acid (ARA, 20:4n-6), and the bioactive n-3 LC-PUFAs are eicosapentaenoic acid (EPA, 20:5n-3) and docosahexaenoic acid (DHA, 20:6n-3). LA and ALA use the same series of enzymes for biosynthesis into their respective LC-PUFAs and therefore, an excess of one decreases the conversion of the other. In the Western diet, the n-6/n-3 ratio is ~16:1; however, for optimal health an n-6/n-3 ratio of 4:1 is recommended [2].

Increased dietary n-3 PUFAs intake promotes retina and brain development in infants [3]. In adults, n-3 PUFA consumption has been reported to improve health by reducing the risk of cardiovascular disease (CVD), obesity, diabetes, inflammation, and several neurological diseases [4]. Atherogenic, pro-thrombotic, and inflammatory effects are influenced by ARA. ARA-derived 2-series eicosanoids synthesized by the enzyme, cyclooxygenase II (COX II), include the platelet aggregating thromboxane B_2 _(TXB_2_), and the pro-inflammatory prostaglandin E_2 _(PGE_2_). EPA using the same COX II enzyme as ARA produces 3-series eicosanoids that are less inflammatory and decrease platelet aggregation. Therefore, increasing EPA intake may competitively inhibit the production of ARA-derived 2-series eicosanoids resulting in decreased inflammation, blood-clotting, and in turn, reduced CVD risk [5].

Reports of numerous health benefits have contributed to the popularity of n-3 PUFA enriched foods and/or supplements [6]. However, different sources of oils provide different types of n-3 PUFAs. Flaxseed oil is a rich source of the n-3 PUFA, ALA, whereas fish oils are rich in the n-3 LC-PUFAs, EPA and DHA. Additionally, various fish oils have different EPA:DHA ratios [7]. Similar to fish oil, krill oil (KO) is rich in EPA and DHA. However, fatty acids from fish oils are mainly associated with triglycerides (TGs), whereas the n-3 PUFAs in KO are associated with phospholipids (PLs) and TGs [8]. PLs and TGs are digested differently and in turn, this may affect n-3 PUFA bioavailability. In human studies, feeding infants DHA in PL form resulted in better absorption than feeding DHA in TG form [3]. Determining the digestibility of n-3 PUFAs provided as PL compared to TG is important because this influences n-3 PUFA incorporation into tissues. Valenzuela et al. [9] reported increased liver DHA accretion in female rats fed n-3 PUFAs in PL compared to TG form. Increasing tissue n-3 PUFA exerts beneficial physiological effects by influencing cell membrane fluidity, membrane-bound receptors, signaling molecules, and gene expression [10]. However, the higher unsaturation due to increased tissue n-3 PUFAs may lead to increased susceptibility to lipid peroxidation and in turn, oxidative stress.

The effect of n-3 LC-PUFA intake on lipid peroxidation has produced inconsistent results of increased oxidation [11-13], no effect on oxidation [14], and even decreased oxidation [15]. PUFAs in PL form have been suggested to have greater stability against lipid oxidation due to their incorporation into cell membranes [16]. However, Song et al. [11] reported that rat fed DHA in PL form resulted in similar oxidation as feeding DHA in TG form. Only when the accumulation of oxidants overwhelms the body's antioxidant enzymes does oxidative stress and negative health effects occur. Therefore, more studies are necessary to determine the effect of different sources of n-3 PUFAs on tissue oxidation and tissue antioxidant defense enzymes.

Currently, various sources of n-3 PUFAs are available with claims that some sources are more beneficial than others. The source of n-3 PUFAs that is most favorable to health should provide high digestibility and efficient tissue incorporation with the least tissue lipid peroxidation. Therefore, the objective of this study was to determine the effect of different sources of n-3 PUFAs on digestibility, tissue deposition, eicosanoid metabolism, and oxidative stability.

## Methods

### Diets

Experimental diets fed to animals were formulated to match the standard purified American Institute of Nutrition-93G (AIN-93 G) diet which meets the nutritional requirements for growing rats as defined by the National Research Council [17]. Modifications of the AIN-93G diet consisted of replacing 7% lipids with 12% lipid by weight. The high fat diet (~27% by kcals) was used to reflect the higher total fat intake typical of the Western diet (~33% by kcals). The dietary oils consisted of either: 1) corn oil (CO), 2) flaxseed oil (FO), 3) krill oil (KO), 4) menhaden oil (MO), 5) salmon oil (SO) or 6) tuna oil (TO). CO, FO, MO, SO, and TO sources were provided by J. Edwards International Inc. (Quincy, MA). KO was purchased from Enzymotec Ltd. (Morristown, NJ). It was necessary to add CO (2 g/kg diet) to the MO and KO (10 g/kg diet) diets to meet the National Research Council [17] nutrient recommendation in rats for the essential n-6 PUFA, linoleic acid (LA, 18:2n-6) (Table 1).

**Table 1:** Whole Diet Composition

	Treatments					
Ingredients (g/kg diet)	CO	FO	KO	MO	SO	TO
ω-3 PUFA Oil Source	0	120	118	118	120	120
Corn Oil	120	0	2	2	0	0
Casein	200	200	200	200	200	200
L-Cystine	3	3	3	3	3	3
Corn Starch	347.5	347.5	347.5	347.5	347.5	347.5
Maltodextrin	132	132	132	132	132	132
Sucrose	100	100	100	100	100	100
Cellulose	50	50	50	50	50	50
Mineral Mix, AIN-93G-MX^1^	35	35	35	35	35	35
Vitamin Mix, AIN-93-VX^1^	10	10	10	10	10	10
Choline Bitartrate	2.5	2.5	2.5	2.5	2.5	2.5

^1 ^Based on the AIN-93G vitamin and mineral mixes [63].

Abbreviations are CO, corn oil; FO, flaxseed oil; KO, krill oil; MO, menhaden oil; SO, salmon oil; TO, tuna oil

Table 2 shows the fatty acid composition and lipid classes of the experimental oil. The lipid sources were selected on the basis that CO is prevalent in the Western diet and provides a high n-6:n-3 ratio of 73:1. FO is the richest source of the essential n-3 PUFA, ALA [18]. The EPA:DHA ratio of 5:1 in SO was higher than 1:2 in TO. KO and MO have a similar EPA:DHA ratio of 3:1; however, fatty acids in MO are in TG form and fatty acids in KO are in PL as well as TG form (Table 2).

**Table 2:** Dietary fatty acids and lipid classes

	Treatments					
**Fatty Acids (mg FA/g Diet)**^**1**^	CO	FO	KO	MO	SO	TO
**n-3 PUFA**	0.1 ± 0.01	14.6 ± 2.1	18.0 ± 4.6	7.7 ± 0.5	12.0 ± 0.8	5.6 ± 0.5
ALA (18:3n-3)	0.1 ± 0.01	14.6 ± 2.1	0.2 ± 0.04	0.2 ± 0.01	0.1 ± 0.01	0.1 ± 0.01
TG^2^	100	100	2.4 ± 0.1	100	100	100
PL^2^	ND	ND	1.8 ± 0.1	ND	ND	ND
EPA (20:5n-3)	ND	ND	13.2 ± 2.8	5.5 ± 0.4	10.0 ± 0.7	2.6 ± 0.3
TG^2^	ND	ND	20.9 ± 0.9	100	100	100
PL^2^	ND	ND	27.2 ± 0.7	ND	ND	ND
DHA (22:6n-3)	ND	ND	4.6 ± 1.9	2.0 ± 0.2	1.9 ± 0.1	2.9 ± 0.2
TG^2^	ND	ND	11.1 ± 0.3	100	100	100
PL^2^	ND	ND	12.8 ± 0.5	ND	ND	ND
**EPA/DHA**	ND	ND	3:1	3:1	5:1	1:2
**n-6 PUFA**	6.4 ± 0.9	5.4 ± 0.80	0.5 ± 0.1	0.2 ± 0.1	0.5 ± 0.1	0.5 ± 0.1
LA (18:2n-6)	6.4 ± 0.9	5.4 ± 0.8	0.5 ± 0.1	0.2 ± 0.1	0.5 ± 0.1	0.2 ± 0.03
TG^2^	100	100	6.9 ± 0.5	100	100	100
PL^2^	ND	ND	2.8 ± 0.03	ND	ND	ND
ARA (20:4n-6)	ND	ND	0.23 ± 0.04	0.16 ± 0.01	0.22 ± 0.01	0.30 ± 0.1
TG^2^	ND	ND	ND	100	100	100
PL^2^	ND	ND	1.4 ± 0.2	ND	ND	ND
**n-6/n-3**	73:1	1:3	1:33	1:48	1:23	1:12

^1^Values are expressed as mean (mg FA/g diet) ± SEM

^2^Values are expressed as mean (%) ± SEM

Abbreviations are CO, corn oil; FO, flaxseed oil; KO, krill oil; MO, menhaden oil; SO, salmon oil; TO, tuna oil; TG, triglyceride; PL, phospholipid; ALA, α-linolenic acid; EPA, eicosapentaenoic acid; DHA, docosahexaenoic acid; LA, linoleic acid; ARA, arachidonic acid; ND, not detectable.

### Animal Feeding Study

All animal procedures were approved by the Animal Care and Use Committee at West Virginia University and were conducted in accordance with the guidelines set forth by the National Research Council Guide for the Care and Use of Laboratory Animals [19]. Growing (28 d) female Sprague-Dawley rats (n = 60) were purchased from Taconic Farms (Rockville, MD). Upon arrival at the West Virginia University animal care facility, rats were individually caged in metabolic cages to determine food intake and to collect urine and fecal samples. Rats were kept housed in rooms maintained at 21°C with a 12 h light/dark cycle throughout the 8 weeks feeding study. Following 7 d acclimation, rats (n = 10/group) were randomly assigned to the experimental diets of CO, FO, KO, MO, SO or TO. Rats were provided 15 ± 0.75 g diet/d of their assigned diet to prevent variability in food intake. This amount was based on the daily average food consumed by growing female Sprague-Dawley rats fed diets containing different sources of n-3 PUFAs [20]. Food intake was measured and fresh diet was provided daily. Water consumption and body weights were measured weekly throughout the 8 week feeding study.

### Determination of Lipid and Fatty Acid Apparent Digestibility

Lipid intake was determined as diet consumed per week × 12% lipid in the diet. Fatty acid intake was determined as diet consumed per week ×% fatty acid in the diet. Lipid apparent digestibility was determined by collecting fecal samples during the final week of the 8 feeding week study. Rats were individually housed in metabolic cages to collect feces. Pooled 7 d fecal samples were freeze-dried (VirTis, Warminster, PA), weighed, and total fecal lipid content determined by Soxhlet extraction [21]. Apparent digestibility of total lipid was measured according to Deuchi et al [22] as [(lipid intake - fecal lipid)/(lipid intake)] × 100. Similarly, apparent digestibility of individual fatty acids was measured using the formula [(fatty acid intake - fecal fatty acids)/(fatty acid intake)] × 100.

### Determination of Fatty Acid Composition

At the end of the 8 weeks, rats were euthanized by CO_2 _inhalation. Brain, liver, retroperitoneal and gonadal adipose tissue was dissected and then weighed. Tissues were immediately frozen in liquid nitrogen and stored at -80°C until analyzed. Lipids were extracted according to Bligh and Dyer [23]. Briefly, aliquots of brain (0.5 g), liver (0.5 g) or adipose tissue (0.025 g) samples were added to Tris/EDTA buffer (pH 7.4) and 48 μl nonadecenoic (19:1) added as an internal standard. Chloroform:methanol:acetic acid (2:1:0.15 v/v/v) solution was added and samples were centrifuged at 900 *g *for 10 min at 10°C. The collected chloroform layer was filtered through 1-phase separation filters. The centrifugation and filtration steps were repeated and the extracted lipid was dried under nitrogen gas. All samples were conducted in duplicate.

The extracted lipid samples were transmethylated according to Fritsche and Johnston [24]. Briefly, fatty acids were methylated by adding 4% sulfuric acid in anhydrous methanol to the extracted lipid samples followed by incubation in a 90°C water bath for 60 min. Samples were cooled to room temperature and 3 mL of deionized distilled water added. Chloroform was added to the methylated samples and centrifuged at 900 *g *for 10 min at 10°C. The collected chloroform layer was filtered through anhydrous sodium sulfate to remove remaining water. Samples were dried under nitrogen gas. Dried samples were diluted in iso-octane to a concentration of 5 mg FAME (fatty acid methyl esters)/mL iso-octane. All samples were conducted in duplicate.

FAME samples were analyzed by gas chromatography (CP-3800, Varian, Walnut Creek, CA) using an initial temperature of 140°C held for 5 min and then increased 1°C per min to a final temperature of 220°C. Total separation time was 60 min. A wall-coated open tubular fused silica capillary column (Varian Inc., Walnut Creek, CA) was used to separate FAMEs with CP-Sil 88 as the stationary phase. Nitrogen was used as the carrier gas. Quantitative 37 Component FAME Sigma Mix (Supelco, Bellefonte, PA) was used as a standard to identify fatty acids. Fatty acids were quantified using peak area counts and retention time.

### Analysis of Lipid Classes

Lipid classes were separated using thin-layer chromatography (TLC) as described in Gigliotti et al. [8]. Briefly, tissue lipid samples were spotted (40 μl) onto Whatman K6F 60Å pore size silica plates containing fluorocein (PJ Cobert Associates, St. Louis, MO). TLC plates were developed using a hexane:ether:acetic acid solution (80:20:1.5 v:v:v) as the mobile phase. The separated lipid classes were visualized using a Fluorochem 8000 densitometer (Alpha Innotech Corp, San Leandro, CA). TLC plate images were photographed using a camera interfaced to the PC and images were analyzed using the spot densitometer Fluorochem program (version 1.0). TGs were identified using the retention factor (R_f_) values obtained from a triolein standard (Sigma-Aldrich, St. Louis, MO). PLs were identified using the R_f _value of soybean lecithin standard (Fisher Scientific, Pittsburgh, PA). Identified lipid classes were scraped from the plates then lipids extracted, methylated, and fatty acid composition determined by gas chromatography according to the methods described above.

### Eicosanoid Measurements

TXB_2 _and PGE_2 _derived from ARA are short-lived molecules. Therefore, the stable metabolites 11-dehydro TXB_2 _and 13, 14-dihydro-15-keto PGE_2 _were measured. Pooled 7 d urine samples were collected during the final week of the 8 wk feeding study. Rats were individually housed in metabolic cages to collect urine. Ascorbic acid (0.1%) was added to the urine collection tubes as a preservative, and mineral oil (1 mL) was added to prevent evaporation. Pooled 7 d urine samples were centrifuged at 1, 500 *g *for 10 min at 4°C. Following centrifugation, urine samples were aliquoted into clean tubes. Urinary 11-dehydro TXB_2 _and 13, 14-dihydro-15-keto PGE_2 _were determined using a commercially available enzyme immunoassay kit according to the manufacturer's instructions (Cayman Chemical, Ann Arbor, MI). Absorbance was determined at wavelength 405 nm using a Spectramax Plus microplate reader (Molecular Devices, Sunnyvale, CA).

### Measurement of Oxidative Stability

The rats were euthanized by CO_2 _inhalation. The chest cavity was opened and the aorta punctured to collect blood. Blood was centrifuged at 1, 500 *g *for 10 min at 4°C to obtain serum. Samples were stored at -80°C until assayed. Serum and liver thiobarbituric acid reactive substances (TBARS) were determined. Liver homogenate was prepared by homogenizing tissue (~0.025 g) in 250 μl of Tris/EDTA buffer (pH 7.4) using a polytron homogenizer. Samples were centrifuged at 1, 500 *g *for 15 min and the supernatant collected. TBARS were measured using a commercially available colorimetric kit (Cayman Chemical, Ann Arbor, MI). Absorbance was determined at 540 nm using a Spectramax Plus microplate reader. All samples were determined in duplicate and TBARS values were expressed as μM/malondialdehyde (MDA).

Total antioxidant capacity (TAC) was measured using a commercially available total antioxidant assay colorimetric assay kit (Cayman Chemical, Ann Arbor, MI). Briefly, serum TAC was determined by diluting serum samples 1:20 v/v with 5 mM potassium phosphate buffer (pH 7.4) containing 0.9% sodium chloride and 0.1% glucose. Liver homogenates were prepared in 1 mL of 5 mM potassium phosphate buffer (pH 7.4). Absorbance was determined at 750 nm using a Spectramax Plus microplate reader. All samples were determined in duplicate and values were expressed as Trolox equivalents.

### RNA Isolation and Gene Expression

Gene expression of antioxidant enzymes was measured by isolating total RNA from liver tissue using the mirVana™ miRNA Isolation Kit (Ambion Inc, Foster City, CA) according to the manufacturer's instructions for total RNA isolation. The concentration of total RNA was quantified using a NanoDrop 1000 spectrophotometer (Thermo Scientific, Waltham, MA). RNA integrity was assessed by agarose gel electrophoresis. First-strand complementary DNA (cDNA) was synthesized using the SuperScript III First-Strand Synthesis System (Invitrogen, Carlsbad, CA), according to the manufacturer's instructions with oligo(dT)_20 _and 600 ng of RNA.

To determine superoxide dismutase (SOD), catalase (CAT), and glutathione-peroxidase (GSH-Px) gene expression, cDNA were amplified in triplicate by RT-qPCR using an iCycler iQ Real-Time PCR Detection System (BioRad, Hercules, CA) in a 5 μl reaction volume using 2.5 μl 2X SYBR Green PCR Master Mix (Applied Biosystems, Carlsbad, CA) with 100 nM of each primer and 1 μL of diluted 1:10 cDNA sample. The primer sequence for Zinc/Copper (Zn/Cu SOD) SOD 1 was (forward 5' - GGT CCA CGA GAA ACA AGA TGA - 3', reverse 5' - CAA TCA CAC CAC AAG CCA AG - 3'), manganese (Mn SOD) SOD 2 was (forward 5' - GAA AGT GCT CAA GAT GGA CAA AG - 3', reverse 5' - CTG AAT GGC TTC CCT GAA TG - 3'), CAT was (forward 5' - TGT TGA ATG AGG AGG AGA GGA - 3', reverse 5' - TTC TTA GGC TTC TGG GAG TTG - 3'), and GSH-Px was (forward 5' - GAT ACG CCG AGT GTG GTT T - 3', reverse 5' - TCT TGA TTA CTT CCT GGC TCC T - 3'). The housekeeping gene GAPDH was used as an internal reference (forward 5' - TCA AGA AGG TGG TGA AGC AG - 3', reverse 5' - CCT CAG TGT AGC CCA GGA TG - 3'). The program used for qRT-PCR amplification consisted of an initial temperature at 50°C for 2 min followed by an initial denaturation for 10 min at 95°C and 40 PCR cycles. Each cycle comprised a melting step at 95°C for 15 sec followed by a joint annealing/extension step at 60°C for 1 min. Specificity of amplification was assessed by a melting curve of each amplicon, and visualization of the expected fragment size on 3% agarose gel. Data were expressed as relative gene expression after normalization to the GAPDH housekeeping gene. The Pfaffl relative quantification model was used for gene expression calculation [25].

### Statistical Analysis

Values were expressed as mean ± standard error of the mean (SEM). One-way analysis of variance (ANOVA) was used to determine differences among treatment groups. Post-hoc multiple comparisons were performed using Tukey's test. Differences were considered significant at *P *< 0.05. Results were analyzed using SigmaStat 3.1 statistical software (Systat Software Inc., San Jose, CA).

## Results

### Food Intake, Body and Tissue Weight

No significant differences were observed for food intake, body weight gain or final body weight among the diet groups (Table 3). Rats fed SO or TO had heavier liver weight (*P *< 0.001) compared to MO, FO or CO-fed rats. KO-fed rats had heavier (*P *< 0.001) liver weights than FO or CO-fed rats. There were no significant differences in brain, gonadal or retroperitoneal adipose tissue weights among the diet groups (Table 3).

**Table 3:** The effect of feeding growing female rats different sources of omega-3 polyunsaturated fatty acids on food intake, body weight, and tissue weights

	Treatments					
**Measurement**^**1**^	CO	FO	KO	MO	SO	TO
Food intake (g)	750.1 ± 12.5	762.9 ± 14.3	767.4 ± 10.9	761.2 ± 17.6	706.8 ± 17.4	738.3 ± 16.4
Body weight gain (g)	76.0 ± 7.4	103.8 ± 8.1	98.4 ± 6.1	107.6 ± 12.5	86.0 ± 8.4	106.0 ± 6.7
Final body weight (g)	214.9 ± 6.4	239.6 ± 8.2	231.7 ± 6.6	241.5 ± 14.4	215.5 ± 10.3	235.0 ± 8.2
Liver weight (g/100 g bwt)	2.9 ± 0.1^c^	2.8 ± 0.1^c^	3.6 ± 0.1^ab^	3.2 ± 0.2^bc^	3.7 ± 0.1^a^	3.8 ± 0.1^a^
Brain weight (g/100 g bwt)	0.8 ± 0.03	0.8 ± 0.03	0.8 ± 0.02	0.8 ± 0.04	0.8 ± 0.04	0.8 ± 0.03
Gonadal adipose weight (g/100 g bwt)	1.1 ± 0.2	1.7 ± 0.2	1.6 ± 0.1	1.8 ± 0.4	1.3 ± 0.2	1.3 ± 0.2
Retroperitoneal adipose weight (g/100 bwt)	0.3 ± 0.04	0.5 ± 0.06	0.4 ± 0.04	0.5 ± 0.07	0.4 ± 0.07	0.3 ± 0.06

^1^Values are expressed as the mean ± SEM of n = 10 rats/group. Different superscript letters a, b, c within the same rows indicate significant differences at *P *< 0.05 by one-way ANOVA followed by Tukey's test. Abbreviations are CO, corn oil; FO, flaxseed oil; KO, krill oil; MO, menhaden oil; SO, salmon oil; TO, tuna oil; bwt, body weight.

### Diet Fatty Acid Content and Apparent Digestibility

Shown in Table 2, KO had the highest total n-3 PUFA content followed by FO. Of the oil sources, FO had the highest ALA content and KO had the highest EPA and DHA content. Of the fish oils, SO had the highest EPA content and TO had the highest DHA content. Dietary n-3 PUFAs were in TG form in FO and fish oil sources. In KO, n-3 PUFAs were approximately equally distributed in TGs and PLs.

Shown in Table 4, apparent ALA digestibility was greater (*P *= 0.005) in rats fed FO compared to TO or CO-fed rats. Apparent ALA digestibility was greater (*P *< 0.001) in KO, MO, and SO than CO-fed rats. No significant differences were observed in the apparent EPA digestibility among rats fed the different marine oils. However, apparent DHA digestibility was higher (*P *= 0.009) in SO than KO-fed rats. There were no significant differences in apparent DHA digestibility in rats fed MO or TO compared to SO or KO-fed rats.

**Table 4:** The effect of feeding growing female rats different sources of omega-3 polyunsaturated fatty acids on apparent digestibility

	Treatments					
**Measurements (%)**^**1**^	CO	FO	KO	MO	SO	TO
Total Lipid	95.9 ± 0.4^c^	96.8 ± 0.3^bc^	93.1 ± 0.7^d^	97.2 ± 0.4^abc^	98.8 ± 0.2^a^	98.0 ± 0.2^ab^
						
**n-3 PUFAs**						
ALA (18:3n-3)	90.2 ± 2.4^c^	99.8 ± 0.02^a^	97.2 ± 0.2^ab^	96.4 ± 0.8^ab^	97.7 ± 0.4^ab^	94.1 ± 0.7^bc^
EPA (20:5n-3)	ND	ND	98.7 ± 0.6	98.8 ± 0.7	98.9 ± 0.6	98.9 ± 0.5
DHA (22:6n-3)	ND	ND	99.4 ± 0.1^b^	99.7 ± 0.1^ab^	99.8 ± 0.04^a^	99.6 ± 0.1^ab^
						
**n-6 PUFAs**						
LA (18:2n-6)	99.4 ± 0.2^a^	99.7 ± 0.03^a^	98.9 ± 0.1^ab^	97.8 ± 0.3^bc^	99.6 ± 0.1^a^	97.5 ± 0.6^c^
ARA (20:4n-6)	ND	ND	97.7 ± 0.3	97.8 ± 0.2	96.8 ± 0.7	96.8 ± 0.6

^1 ^Values are expressed as the mean ± SEM of n = 10 rats/group. Different superscript letters a, b, c, d within the same rows indicate significant differences at *P *< 0.05 by one-way ANOVA followed by Tukey's test. Abbreviations are CO, corn oil; FO, flaxseed oil; KO, krill oil; MO, menhaden oil; SO, salmon oil; TO, tuna oil; PUFA, polyunsaturated fatty acids; ALA, α-linolenic acid; EPA, eicosapentaenoic acid; DHA, docosahexaenoic acid; LA, linoleic acid; ARA, arachidonic acid; ND, not detectable.

Shown in Table 2, dietary n-6 PUFAs were in TG form in FO and fish oil sources. In KO, the n-6 PUFA, LA was predominantly in the TG form, whereas ARA was in PL form. Shown in Table 4, apparent LA digestibility was lower (*P *< 0.001) in TO-fed rats than all groups, except MO. Apparent LA digestibility was significantly lower in MO than SO, FO or CO-fed rats. No significant differences were observed in apparent ARA digestibility among the diet groups. Apparent digestibility of total lipids was lowest (*P *< 0.02) in rats fed KO. Apparent digestibility of total lipids was greater (*P *= 0.008) in SO and TO than CO-fed rats. SO fed rats also had greater (*P *= 0.02) apparent total lipid digestibility than FO-fed rats

### Tissue Deposition

#### Brain Fatty Acid Profile

The major lipid class in the brain was PL (54.2-63.1%) with the remaining portion consisting of polar non-PLs (36.9-45.8%). SO and TO-fed rats had the highest (*P *< 0.001) brain DHA deposition. Rats fed KO had significantly higher brain DHA than FO-fed rats. There were no significant differences in brain DHA deposition in rats fed MO compared to KO, FO or CO-fed rats. Brain EPA deposition was lower (*P *= 0.01) in rats fed TO compared to MO and FO-fed rats. Brain EPA was not detectable in CO-fed rats. Brain ALA deposition was not significantly different among the diet groups. Overall, rats fed SO and TO had the highest (*P *< 0.007) brain n-3 PUFA deposition (Table 5).

**Table 5:** The effect of feeding growing female rats different sources of omega-3 polyunsaturated fatty acids on brain fatty acid profile

	Treatments					
**Fatty Acid Measurement**^**1**^	CO	FO	KO	MO	SO	TO
**n-3 PUFA**	4.7 ± 0.5^b^	4.1 ± 0.3^b^	5.6 ± 0.5^b^	5.3 ± 0.3^b^	9.2 ± 0.5^a^	7.7 ± 0.4^a^
ALA (18:3n-3)	0.4 ± 0.05	0.3 ± 0.07	0.4 ± 0.1	0.4 ± 0.1	0.5 ± 0.1	0.5 ± 0.1
EPA (20:5n-3)	ND	0.4 ± 0.1^a^	0.2 ± 0.03^ab^	0.4 ± 0.1^a^	0.2 ± 0.02^ab^	0.1 ± 0.003^b^
DHA (22:6n-3)	4.3 ± 0.5^bc^	3.4 ± 0.2^c^	5.0 ± 0.5^b^	4.5 ± 0.1^bc^	8.5 ± 0.5^a^	7.2 ± 0.3^a^
						
**n-6 PUFA**	3.7 ± 0.4^a^	2.2 ± 0.1^bc^	2.3 ± 0.1^bc^	2.1 ± 0.1^c^	3.4 ± 0.1^a^	3.0 ± 0.1^b^
LA (18:2n-6)	0.4 ± 0.03^a^	0.3 ± 0.02^a^	0.2 ± 0.02^b^	0.2 ± 0.01^b^	0.1 ± 0.005^c^	0.05 ± 0.004^c^
ARA (20:4n-6)	3.3 ± 0.4^a^	1.9 ± 0.1^b^	2.1 ± 0.1^b^	2.0 ± 0.1^b^	3.4 ± 0.1^a^	2.9 ± 0.1^a^

^1^Values are expressed as mean (mg FA/g tissue) ± SEM of n = 10 rats/group. Different superscript letters a, b, c within the same rows indicate significant differences at *P *< 0.05 by one-way ANOVA followed by Tukey's test. Abbreviations are CO, corn oil; FO, flaxseed oil; KO, krill oil; MO, menhaden oil; SO, salmon oil; TO, tuna oil; PUFA, polyunsaturated fatty acids; ALA, α-linolenic acid; EPA, eicosapentaenoic acid; DHA, docosahexaenoic acid; LA, linoleic acid; ARA, arachidonic acid; ND, not detectable.

Regarding n-6 PUFAs, brain LA deposition was highest (*P *< 0.001) in CO and FO-fed rats. Brain LA deposition was also higher (*P *= 0.007) in KO and MO than SO or TO-fed rats. Brain ARA deposition was significantly higher (*P *< 0.03) in SO, TO or CO compared to FO, MO and KO-fed rats. Overall, total n-6 PUFA content in the brain was significantly lower in MO, FO, TO, and KO than SO or CO-fed rats (Table 5).

#### Liver Fatty Acid Profile

Shown in Table 6, total n-3 PUFA deposition in the liver was highest (*P *< 0.001) in SO and TO-fed rats. Liver content of specific n-3 PUFAs was also evaluated. Liver ALA deposition was highest (*P *< 0.001) in FO-fed rats with ALA stored mainly as TGs. Of the n-3 LC-PUFAs, liver EPA deposition was highest (*P *< 0.001) in SO-fed rats. EPA was not detectable in the liver of CO-fed rats. FO had lower (*P *= 0.02) liver EPA-TG compared to KO, MO or SO-fed rats. FO and KO-fed rats had highest (*P *= 0.002) liver EPA-PL. Rats fed SO and TO had the highest (*P *< 0.001) liver DHA deposition. In rats fed fish oil, DHA was mainly stored in TG (18-23%) compared to PL (13-16%). In KO-fed rat, liver DHA was almost equally stored in TGs (14.9 ± 0.6%) and PLs (15.5 ± 0.5%).

**Table 6:** The effect of feeding growing female rats different sources of omega-3 polyunsaturated fatty acids on liver fatty acid profile

	Treatments					
**Fatty Acid Measurement**^**1**^	CO	FO	KO	MO	SO	TO
**n-3 PUFA**	0.9 ± 0.1^b^	7.8 ± 1.0^b^	9.6 ± 1.3^b^	9.5 ± 1.1^b^	43.6 ± 5.8^a^	35.9 ± 2.5^a^
**ALA (18:3n-3)**	0.1 ± 0.01^b^	4.4 ± 0.7^a^	0.1 ± 0.01^b^	0.3 ± 0.1^b^	0.3 ± 0.1^b^	0.2 ± 0.04^b^
TG^2^	0.4 ± 0.03^b^	21.0 ± 1.6^a^	0.8 ± 0.1^b^	1.7 ± 0.2^b^	1.0 ± 0.1^b^	0.7 ± 0.1^b^
PL^2^	0.1 ± 0.03^b^	0.9 ± 0.1^a^	0.3 ± 0.03^b^	0.3 ± 0.2^b^	0.1 ± 0.05^b^	0.1 ± 0.05^b^
**EPA (20:5n-3)**	ND	1.7 ± 0.2^b^	3.7 ± 0.5^b^	3.0 ± 0.6^b^	19.4 ± 3.1^a^	6.3 ± 0.6^b^
TG^2^	ND	2.9 ± 0.1^c^	7.5 ± 0.8^a^	6.8 ± 0.6^ab^	7.3 ± 0.8^a^	4.0 ± 0.5^bc^
PL^2^	ND	8.9 ± 1.0^a^	7.2 ± 0.7^a^	3.7 ± 0.6^bc^	4.3 ± 0.3^b^	2.0 ± 0.1^c^
**DHA (22:6n-3)**	0.9 ± 0.1^b^	1.7 ± 0.2^b^	5.7 ± 0.8^b^	6.2 ± 0.7^b^	23.8 ± 2.9^a^	29.4 ± 2.0^a^
TG^2^	1.0 ± 0.1^c^	1.0 ± 0.1^c^	14.9 ± 0.6^b^	18.2 ± 1.1^ab^	20.2 ± 1.1^ab^	23.5 ± 1.6^a^
PL^2^	5.2 ± 0.7^c^	9.5 ± 0.6^bc^	15.5 ± 0.5^ab^	13.7 ± 2.9^ab^	13.7 ± 0.9^ab^	16.1 ± 0.9^a^
**n-6 PUFA**	11.9 ± 0.9^a^	5.9 ± 0.9^b^	3.0 ± 0.1^c^	2.7 ± 0.3^c^	6.7 ± 0.6^b^	7.6 ± 0.5^b^
**LA (18:2n-6)**	7.1 ± 0.7^a^	4.1 ± 0.7^b^	1.4 ± 0.1^c^	1.3 ± 0.2^c^	2.0 ± 0.4^c^	1.6 ± 0.2^c^
TG^2^	35.8 ± 1.6^a^	15.6 ± 0.5^b^	5.9 ± 0.4^c^	5.7 ± 0.5^c^	5.0 ± 0.4^c^	4.2 ± 0.2^c^
PL^2^	9.9 ± 0.8^a^	11.7 ± 0.6^a^	3.4 ± 0.^b^	3.2 ± 0.4^b^	2.7 ± 0.3^b^	2.3 ± 0.3^b^
**ARA (20:4n-6)**	4.8 ± 0.3^b^	1.9 ± 0.3^c^	1.6 ± 0.1^c^	1.4 ± 0.1^c^	4.7 ± 0.3^b^	6.0 ± 0.4^a^
TG^2^	4.3 ± 0.5^a^	0.6 ± 0.1^c^	0.8 ± 0.1^c^	1.2 ± 0.2^bc^	1.4 ± 0.1^bc^	2.1 ± 0.2^b^
PL^2^	26.0 ± 0.9^a^	12.4 ± 0.7^bc^	9.4 ± 0.5^c^	10.0 ± 1.9^c^	11.7 ± 0.7^c^	15.3 ± 0.7^b^

^1^Values are expressed as mean (mg FA/g tissue) ± SEM of n = 10 rats/group.

^2^Values are expressed as mean (%) ± SEM of n = 10 rats/group.

Different superscript letters a, b, c within the same rows indicate significant differences at *P *< 0.05 by one-way ANOVA followed by Tukey's test. Abbreviations are CO, corn oil; FO, flaxseed oil; KO, krill oil; MO, menhaden oil; SO, salmon oil; TO, tuna oil; PUFA, polyunsaturated fatty acids; ALA, α-linolenic acid; EPA, eicosapentaenoic acid; DHA, docosahexaenoic acid; LA, linoleic acid; ARA, arachidonic acid; TG, triglyceride; PL, phospholipid; ND, not detectable.

Evaluation of specific n-6 PUFAs showed rats fed FO or marine oils had lower (*P *< 0.001) liver LA deposition than CO-fed rats. Rats fed marine oils had lower (*P *= 0.01) liver LA deposition than FO-fed rats. Similarly, liver LA-TG and PL was significantly decreased in rats fed marine oils compared to FO or CO-fed rats. The n-6 LC-PUFA, liver ARA was mainly stored in PL form (9.4-26%) rather than TG form (0.6-4.3%) in all diet groups. Rats fed TO (*P *< 0.001) had the highest liver ARA deposition. Rats fed FO, MO and KO had lower (*P *< 0.001) liver ARA than TO, SO or CO-fed rats. Liver total n-6 PUFA deposition in the liver was lower (*P *< 0.001) in rats fed FO or marine oils compared to CO-fed rats. Additionally, MO and KO-fed rats had lower (*P *= 0.01) total n-6 PUFA deposition than FO, SO or TO-fed rats (Table 6).

#### Adipose Fatty Acid Profile

Fatty acids were predominantly in TG form in adipose tissues (data not shown). In gonadal adipose tissue, ALA deposition was highest (*P *< 0.001) in FO-fed rats. EPA deposition was highest (*P *= 0.04) in KO-fed rats. Neither EPA nor DHA were detectable in CO-fed rats. EPA and DHA were detectable in the gonadal adipose tissue of rats fed FO. DHA deposition was higher (*P *< 0.001) in KO, MO, and TO than FO-fed rats. Rats fed MO and TO also had higher (*P *= 0.03) DHA deposition than SO-fed rats. Total n-3 PUFA deposition was highest (*P *< 0.001) in FO-fed rats. KO and MO-fed rats had higher (*P *= 0.002) total n-3 PUFA deposition than CO-fed rats.

TO-fed rats had the highest (*P *= 0.03) ARA deposition. LA and total n-6 PUFA deposition was lower (*P *< 0.001) in rats fed FO and marine oils compared to CO-fed rats. Rats fed marine oils also had lower (*P *< 0.001) LA and total n-6 PUFA deposition than FO-fed rats (Table 7).

**Table 7:** The effect of feeding growing female rats different sources of omega-3 polyunsaturated fatty acids on gonadal and retroperitoneal adipose tissue fatty acid profile

	Treatments					
**Fatty Acid Measurement**^**1**^	CO	FO	KO	MO	SO	TO
***Gonadal Fat Pad***						
**n-3 PUFA**	1.3 ± 0.2^c^	81.7 ± 6.8^a^	21.5 ± 2.6^b^	21.6 ± 2.0^b^	9.5 ± 2.8^bc^	13.5 ± 2.4^bc^
ALA (18:3n-3)	1.3 ± 0.2^b^	80.9 ± 6.6^a^	3.1 ± 0.4^b^	5.0 ± 0.4^b^	1.1 ± 0.2^b^	2.0 ± 0.4^b^
EPA (20:5n-3)	ND	0.7 ± 0.2^c^	9.7 ± 1.3^a^	5.6 ± 0.9^b^	4.2 ± 1.4^bc^	1.9 ± 0.4^bc^
DHA (22:6n-3)	ND	0.07 ± 0.02^c^	8.7 ± 1.2^ab^	10.9 ± 1.0^a^	4.2 ± 1.2^bc^	9.7 ± 2.0^a^
**n-6 PUFA**	107.9 ± 14.1^a^	59.8 ± 5.8^b^	17.0 ± 2.0^c^	21.0 ± 1.8^c^	8.0 ± 1.4^c^	11.6 ± 2.2^c^
LA (18:2n-6)	106.5 ± 13.9^a^	59.4 ± 5.6^b^	15.5 ± 1.9^c^	19.5 ± 1.7^c^	6.5 ± 1.3^c^	8.5 ± 1.6^c^
ARA (20:4n-6)	1.4 ± 0.2^b^	0.5 ± 0.1^b^	1.6 ± 0.2^b^	1.5 ± 0.1^b^	1.4 ± 0.3^b^	3.1 ± 0.7^a^
						
***Retroperitoneal Fat Pad***						
**n-3 PUFA**	2.9 ± 0.2^c^	149.4 ± 15.1^a^	45.7 ± 6.9^b^	37.9 ± 5.1^b^	20.4 ± 4.3^bc^	23.1 ± 3.6^bc^
ALA (18:3n-3)	2.9 ± 0.2^b^	148.4 ± 15.1^a^	5.0 ± 0.6^b^	8.0 ± 0.7^b^	2.7 ± 0.3^b^	2.8 ± 0.6^b^
EPA (20:5n-3)	ND	1.0 ± 0.2^c^	19.9 ± 3.1^a^	10.8 ± 1.6^b^	10.5 ± 2.3^b^	3.2 ± 0.7^bc^
DHA (22:6n-3)	ND	ND	20.7 ± 3.9^a^	23.3 ± 2.1^a^	9.4 ± 1.9^b^	17.0 ± 2.8^ab^
**n-6 PUFA**	211.3 ± 25.4^a^	97.7 ± 10.3^b^	34.4 ± 4.9^c^	33.6 ± 5.0^c^	14.8 ± 2.8^c^	13.9 ± 1.8^c^
LA (18:2n-6)	208.9 ± 25.2^a^	97.2 ± 10.3^b^	32.1 ± 4.9^c^	34.4 ± 3.4^c^	14.6 ± 2.5^c^	11.4 ± 1.7^c^
ARA (20:4n-6)	2.4 ± 0.3^ab^	0.5 ± 0.1^c^	2.3 ± 0.3^ab^	2.9 ± 0.2^a^	1.8 ± 0.2^b^	2.5 ± 0.2^ab^

^1^Values are expressed as mean (mg FA/g tissue) ± SEM of n = 10 rats/group. Different superscript letters a, b, c within the same rows indicate significant differences at *P *< 0.05 by one-way ANOVA followed by Tukey's test. Abbreviations are CO, corn oil; FO, flaxseed oil; KO, krill oil; MO, menhaden oil; SO, salmon oil; TO, tuna oil; PUFA, polyunsaturated fatty acids; ALA, α-linolenic acid; EPA, eicosapentaenoic acid; DHA, docosahexaenoic acid; LA, linoleic acid; ARA, arachidonic acid; ND, not detectable.

In retroperitoneal adipose tissue, ALA deposition was highest (*P *< 0.001) in FO-fed rats. EPA deposition was highest (*P *< 0.001) in KO-fed rats. EPA was not detectable in CO-fed rats. Rats fed FO had lower (*P *= 0.006) EPA deposition than KO, MO or SO-fed rats. DHA was not detectable in the retroperitoneal adipose of CO or FO-fed rats. DHA deposition was higher (*P *< 0.04) in KO and MO than SO-fed rats. Overall, total n-3 PUFA deposition was highest (*P *< 0.001) in FO-fed rats. Rats fed KO or MO had greater (*P *= 0.001) total n-3 PUFA deposition than CO-fed rats.

Regarding n-6 PUFA deposition, retroperitoneal adipose tissue LA deposition was lower (*P *< 0.001) in rats fed FO and marine oils compared to CO-fed rats. Rats fed marine oils had lower (*P *= 0.006) LA deposition compared to FO-fed rats. Retroperitoneal adipose tissue ARA was lowest (*P *< 0.001) in FO-fed rats. Total n-6 PUFA deposition was lower (*P *< 0.001) in rats fed FO and marine oils compared to CO-fed rats. Rats fed marine oils also had lower (*P *= 0.01) total n-6 PUFA deposition than FO-fed rats (Table 7).

#### Eicosanoids Production and Oxidative Stability

**Figure 1:**
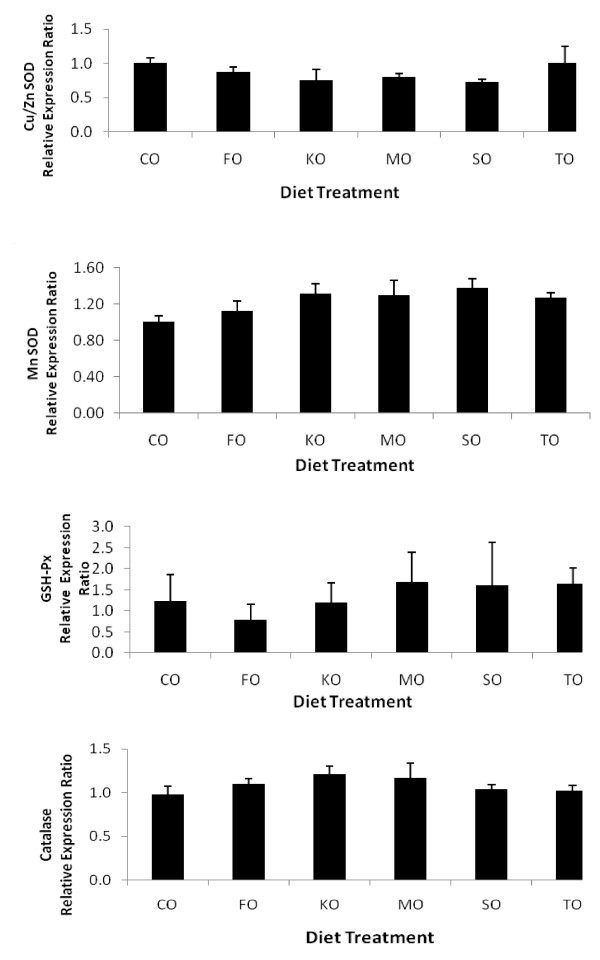
**The effect of feeding growing female rats different sources of omega-3 polyunsaturated fatty acids on gene expression of A) glutathione-peroxidase (GSH-Px), B) copper/zinc superoxide dismutase (Cu/Zn SOD1), C) manganese superoxide dismutase (Mn SOD2), and D) catalase**. Values are relative mRNA expression after normalization to the GADPH housekeeping gene expressed as the means ± SEM (n = 4-6). Abbreviations for diet treatments are CO, corn oil; FO, flaxseed oil; KO, krill oil; MO, menhaden oil; SO, salmon oil; TO, tuna oil.

There were no significant differences in urinary 13, 14-dihydro-15-keto PGE_2 _or 11-dehydro-TXB_2 _among the diet groups (Table 8). There were no differences in RBC TBARS among the diet groups. Serum TBARS were lower (*P *= 0.005) in SO and TO than CO, KO or MO-fed rats. However, there were no differences in serum TAC among the treatment groups. Liver TBARS were highest (*P *= 0.03) in MO-fed rats. Rats fed MO also had greater (*P *= 0.03) liver TAC than KO, SO or TO-fed rats (Table 8). There were no significant differences in relative gene expression of Zn/Cu SOD (Figure 1A), Mn SOD (Figure 1B), CAT (Figure 1C) or GSH-Px (Figure 1D) among the dietary treatment groups.

**Table 8:** The effect of feeding growing female rats different sources of omega-3 fatty acids on oxidative stability and eicosanoid metabolism

		Treatments				
**Measurements**^**1**^	CO	FO	KO	MO	SO	TO
***Eicosanoids***						
PGE_2 _metabolite (μg/d)	2.2 ± 1.5	1.7 ± 1.1	4.6 ± 2.1	0.9 ± 0.04	1.3 ± 1.0	1.1 ± 0.3
TXB_2 _metabolite (μg/d)	1474 ± 665	991 ± 393	1653 ± 447	807 ± 248	1972 ± 808	1359 ± 431
						
***Oxidative Stability***						
RBC TBARS (μM MDA/mL)	9.5 ± 0.6	9.2 ± 0.60	8.5 ± 0.2	9.4 ± 0.5	10.0 ± 0.6	9.6 ± 0.2
Serum TBARS (μM MDA/mL)	6.9 ± 0.4^a^	5.9 ± 0.2^ab^	6.5 ± 0.3^a^	6.8 ± 0.3^a^	5.1 ± 0.3^b^	5.2 ± 0.2^b^
Serum TAC(mM Trolox)	1.5 ± 0.2	1.1 ± 0.1	1.3 ± 0.1	1.5 ± 0.2	1.1 ± 0.2	1.2 ± 0.2
Liver TBARS (μM MDA/g Tissue)	220.3 ± 23.8^b^	235.6 ± 21.7^b^	265.1 ± 46.4^b^	435.0 ± 68.8^a^	198.3 ± 23.3^b^	177.5 ± 14.7^b^
Liver TAC (mM Trolox)	3832 ± 421^ab^	4203 ± 207^ab^	3542 ± 726^b^	5695 ± 727^a^	2726 ± 250^b^	2493 ± 195^b^

^1^Values are expressed as the mean ± SEM of n = 7 rats/group. Different superscript letters a, b within the same rows indicate significant differences at *P *< 0.05 by one-way ANOVA followed by Tukey's test. Abbreviations are CO, corn oil; FO, flaxseed oil; KO, krill oil; MO, menhaden oil; SO, salmon oil; TO, tuna oil; PGE_2 _, prostaglandin E_2_; TXB_2_, thromboxane B2; RBC, red blood cells; TBARS, thiobarbituric acid reactive substances; MDA, malondialdehyde; TAC, total antioxidant capacity.

## Discussion

The variety of commercially available n-3 PUFA sources raises the question of which is the most beneficial. In KO, PUFAs are mainly in PL form whereas in fish oils, PUFAs are mainly in TG form, leading to claims that n-3 LC-PUFAs are better absorbed as KO than fish oils [26]. This study compared sources of n-3 PUFAs that differed in the amount, type, and/or structural form of n-3 PUFAs.

### Apparent Digestibility

PUFA digestibility was influenced by the structural form. In our study, DHA content was ~2-4 times higher in KO compared to the other sources of n-3 PUFAs. However, apparent digestibility was greater (*P *= 0.009) in SO compared to KO-fed rats. This may be due to ~50% of DHA being in PL form in KO, whereas DHA was entirely in TG form in SO. DHA was also in TG form in the other fish oils (MO and TO), yet apparent DHA digestibility was not significantly different than KO-fed rats. In human studies, absorption efficiency of DHA from KO was similar to MO [27,28]. In our study, higher DHA digestibility in SO, but not rats fed MO or TO may be due to differences in the positional distribution of DHA on the TG molecule. Christensen et al. [29] reported DHA on the sn-2 position resulted in higher absorption compared to the sn-1 and sn-3 position of the TG molecule. While, the fatty acid position on the lipid structure may be a factor affecting digestibility. Overall total lipid digestibility was lowest (*P *< 0.02) in KO-fed rats which may be accounted for by fatty acids in KO being associated with PL. Amate and Ramirez [30] observed reduced absorption of n-3 LC-PUFAs in rats fed PL-rich pig brain. In contrast, infants and pre-term infants fed LC-PUFA PL as egg lecithin improved fat absorption [31,32]. In the brain, DHA are mainly associated with phosphatidylethanolamine and phosphatidylserine [33], whereas the predominant PL in egg is phosphatidylcholine [34]. Further studies are needed to clarify whether different PLs influence fatty acid digestibility. This is important because the source of PUFAs that provides high digestibility also increases tissue deposition.

### Brain PUFA Deposition

Epidemiological studies have linked low DHA to poor neural development in infants and to cognitive decline in the aging individuals [35]. In animals, decreased DHA in the developing brain resulted in deficits in neurogenesis, neurotransmitters, visual function, and learning [35]. In humans, PL contributes to ~25% of the dry weight of the brain [36]. However, in our study DHA was better incorporated when consumed in TG than PL form. TO with the highest DHA in TG form resulted in the highest (*P *< 0.001) brain DHA deposition. KO with the highest DHA in PL form did not result in the highest brain deposition due to reduced DHA digestibility. The higher DHA digestibility associated with SO also resulted in the highest (*P *< 0.001) brain DHA deposition. Talahalli et al. [37] observed rats fed EPA+ DHA increased brain DHA, but produced only a small increase in EPA due to inefficient tissue uptake. Of the marine oils, TO with the lowest EPA content resulted in lower (*P *= 0.01) brain EPA deposition compared to rats fed MO or FO. Dietary FO contained no detectable EPA; therefore, EPA deposition in the brain tissue of FO-fed rats suggested that *de novo *synthesis of the n-3 LC-PUFAs occurred in the brain.

Brain metabolism, function, and structure also depend on adequate concentrations of ARA [38]. In our study, the CO and FO diets had no detectable ARA, but the highest LA content. In turn, brain LA content was highest (*P *< 0.001) in CO and FO-fed rats. Efficient metabolism of LA to n-6 LC-PUFAs in the brain was indicated by ARA deposition in rats fed CO and FO. Brain ARA content in CO-fed rats was comparable to rats fed SO and TO with the highest dietary ARA-TG content. On the other hand, conversion of ALA to n-3 LC-PUFAs was less efficient. Feeding rats FO, but not CO, increased brain EPA. This may be due to higher ALA content in the FO (14.6 ± 2.1 mg/g) than the CO (0.1 ± 0.01 mg/g) diet. Brain DHA deposition in rats fed CO and FO diets containing no DHA indicated conversion of ALA to n-3 LC-PUFAs. Several studies reported increased brain DHA in rats fed ALA [39-42]. In our study, rats fed SO and TO with pre-formed DHA had significantly higher brain DHA deposition than FO or CO-fed rats having no dietary DHA. The results indicated greater brain incorporation of pre-formed DHA compared to conversion of ALA to n-3 LC-PUFAs. KO and MO also provided pre-form n-3 LC-PUFAs; however, DHA content provided by MO was low and KO had reduced DHA digestibility. Due to limited biosynthesis of DHA in the mammalian brain, DHA deposition in the brain relies on the diet and on the release of DHA synthesized from ALA in the liver [33,43]. Therefore, the effect of feeding different sources of n-3 PUFA on liver fatty acid composition was evaluated.

### Liver PUFA Deposition

The amount of dietary fatty acids affected liver deposition. Rats fed FO with the highest ALA content also had the highest (*P *< 0.001) liver ALA deposition. Rats fed SO with the highest dietary EPA-TG resulted in the highest (*P *< 0.001) liver EPA deposition. Of the oil sources, KO had the highest DHA content; however, liver DHA deposition was highest (*P *< 0.001) in rats fed TO and SO. TO provided the highest DHA in TG form and SO-fed rats showed greater DHA digestibility than KO. Liver DHA incorporation as TO was ~1.5 times greater in the form of TG than PL in TO and SO-fed rats, whereas DHA was equally incorporated as TG and PL in KO-fed rats. Song and Miyazawa [12] reported that rats fed DHA in PL form had lower liver DHA incorporation compared to DHA fed in TG form.

The liver is a major site of LC-PUFA biosynthesis. Therefore, n-3 LC-PUFA deposition is not only dependent on the intake of pre-formed EPA and DHA, but it also depends on intake of the precursor, ALA. Feeding rats CO containing ALA and no n-3 LC-PUFAs resulted in DHA, but no detectable EPA liver deposition. DHA has a structural role, whereas EPA is preferentially utilized for β-oxidation or eicosanoid synthesis [44]. Similarly, FO contains no pre-formed n-3 LC-PUFA. Feeding rats FO with the highest ALA content resulted in detectable DHA as well as EPA deposition in the liver. Still, conversion of ALA to n-3 LC-PUFAs resulted in lower liver EPA and DHA deposition compared to consumption of SO and TO with pre-formed n-3 LC-PUFAs. Talahalli et al. [37] reported rats needed to consume 12.5 times more ALA to produce the same liver n-3 LC-PUFAs incorporation as rats consuming EPA and DHA. This may be due to preferential use of ALA in β-oxidation [45]. Also, several enzymes required for synthesis of LC-PUFAs in the desaturation-elongation pathway are inefficient [46].

On the other hand, there was efficient liver conversion of LA to n-6 LC-PUFAs. This was indicated by similar liver ARA deposition in rats fed CO containing no detectable ARA compared to SO containing pre-formed ARA. Additionally, liver ARA deposition was significantly higher in CO than FO, KO or MO-fed rats. This may have occurred because FO had the highest ALA content followed by MO and KO. ALA and LA compete for the same enzymes to form their respective LC-PUFAs with ALA having higher affinity for the rate-limiting Δ-6 desaturase [33]. This has important implications because decreased ARA reduces synthesis of pro-thrombotic and inflammatory 2-series eicosanoids by COX II. However, our study showed no significant differences in the 2-series eicosanoids, PGE_2 _and TXB_2 _metabolites among the diet groups. Feeding rats FO, which is high in ALA, decreased liver ARA, but this was not accompanied by increased EPA deposition. Conversely, feeding rats SO increased liver EPA, but this was not accompanied by decreased ARA deposition. Furthermore, measurement of urinary PGE_2 _and TXB_2 _metabolites provided an indicator of systemic rather than tissue change. Responses to n-3 PUFA intakes are not uniform among tissues. For example, liver fatty acid composition may vary depending on the uptake of chylomicron remnants from the circulation [47]. Also, the liver not being a major storage organ is less responsive to the diet than adipose tissues [37]. Recent research suggests changing the fatty acid profile of adipose tissue results in health benefits by altering lipid metabolism and adipokine secretion [48,49]. Therefore, the effect of consuming different n-3 PUFA sources on adipose tissue PUFA composition was determined.

### Adipose Tissues PUFA Deposition

As the chief site for lipid storage, the fatty acid profile of adipose tissue reflected the diet. Rats fed KO with the highest amount of EPA had the highest (*P *< 0.001) adipose tissue EPA deposition. Rats fed FO with the highest amount of ALA content had the highest (*P *< 0.001) adipose tissue ALA deposition. Talahalli et al [37] reported rats fed increasing doses of ALA resulted in a linear increase in ALA accumulation in the adipose tissue. In our study, rats fed FO increased n-3 PUFA deposition in the adipose due to the high dietary ALA content. Additionally, rats fed KO and MO increased adipose n-3 PUFA deposition. Others also reported feeding rats fish oils increased n-3 LC-PUFA incorporation in the adipose tissue [50-53]. Increasing adipose tissue n-3 PUFA incorporation has been observed to reduce adipose mass [48]. However, in our study, there was no significant reduction in the adipose mass of rats fed different n-3 PUFA sources.

The adipose tissue is another site of LC-PUFA biosynthesis. Efficient conversion of LA to n-6 LC-PUFAs in gonadal adipose was indicated by rats fed FO and CO containing no ARA having similar tissue ARA deposition to rats fed MO, KO or SO containing pre-formed ARA. In retroperitoneal adipose, rats fed CO had similar tissue ARA to rat fed marine oils. FO also resulted in tissue ARA, but lower (*P *< 0.001) amounts than rats fed CO or marine oils. This may be due to FO having the highest ALA content. High ALA competitively inhibits LA, the precursor of ARA.

Conversion of ALA to n-3 LC-PUFAs was less efficient. In our study, there were no detectable EPA and DHA in the adipose of CO-fed rats. However, rats fed FO containing ALA, but no DHA, resulted in DHA deposition in gonadal and not in retroperitoneal adipose tissue. This suggested conversion of ALA to n-3 LC-PUFAs was less efficient in retroperitoneal adipose compared to gonadal adipose. Muhlhausler et al. [49] observed female rats fed n-3 PUFA had different degrees of responsiveness to PUFA deposition and in turn, tissue PUFAs induced changes in lipogenic gene expression. Furthermore, gene expression changes were more pronounced in the omental compared to retroperitoneal adipose tissue.

Rats fed FO and marine oils had an adipose tissue n-6/n-3 ratio of 1:1-1:2. An n-6/n-3 ratio of ~1:1 in tissues has been reported to reduce atherosclerosis due to the inhibition of systemic and vascular inflammation in apolipoprotein E-deficient mice [54]. While the optimal n-6/n-3 ratio in tissues has not been defined, increasing tissue unsaturation has been considered to be health beneficial. However, the higher tissue unsaturation needs to be considered since a greater number of double bonds increases tissue susceptibility to lipid peroxidation.

### Oxidative Stability

DHA is particularly susceptible to lipid peroxidation due to its high degree of unsaturation [11]. Our study showed rats fed TO, the highest source of DHA of the fish oils or SO with the high DHA digestibility resulted in lower (*P *= 0.005) serum TBARS compared to all diet groups, except FO-fed rats. However, there were no significant differences in RBC TBARS among the diet groups. Oxidative stress occurs when accumulation of oxidative products overwhelms the body's antioxidant capacity. TAC measures endogenous antioxidant, dietary antioxidant, and interactions between antioxidants [55]. In our study, serum TAC was not significantly different among the diet groups. Circulating TBARS and TAC may not reflect the tissue concentration.

The liver is a primary target for oxidative stress-induced damage in oil-fed rats [12]. Rats fed MO had the highest (*P *= 0.03) liver TBARS. The TBARS assay is accepted as an index of oxidative stress; however, this method quantifies MDA-like compounds and does not specifically measure lipid peroxidation [56]. Furthermore, it is the imbalance of oxidants and antioxidants that leads to oxidative stress. Reena and Lokesh [57] reported that the lipid peroxides generated by feeding rats PUFAs was partly nullified by the capability of PUFAs to increased liver antioxidant enzymes, Cu/Zn SOD, Mn SOD, CAT, and GSH-Px. Extracellular and cytosolic forms of SOD depend on Cu/Zn and in the mitochondria SOD depends on Mn. SOD detoxifies superoxide radicals giving rise to hydrogen peroxide (H_2_O_2_). H_2_O_2 _is a potent free radical generator and can generate hydroxyl radicals, which induce lipid peroxidation of cell membranes. Therefore, to prevent accumulation of H_2_O_2_, it is important that enhanced SOD activity be accompanied by increased cellular CAT and GSH-Px [58]. Ruiz-Gutierrez et al. [59] reported increased n-3 PUFAs enhanced the efficiency of the antioxidant defense system.

In our study, rats fed SO and TO had a P:S ratio of 3:1 compared the range of 2:1 to 1.2:1 in the other treatment groups. However, liver TAC was higher in MO (*P *= 0.03) than TO or SO-fed rats. According to Venkatraman et al. [60], different lipids had differential effects on the antioxidant defense system at the molecular level. Mice fed MO and KO had higher liver SOD, CAT, and GSH-Px mRNA expression than rats fed CO [60]. In our study, there were no significant differences in SOD or CAT gene expression in rats fed different sources of n-3 PUFAs. Rats fed fish oils increased GSH-Px mRNA expression compared to CO or FO-fed rats, although this was not statistically significant. Demoz et al. [15] reported that mice provided high doses of EPA enhanced liver antioxidant enzyme activities. In our study, EPA was in TG form in SO, whereas in KO ~27% of EPA was in PL form. In turn, liver deposition of EPA as PL was ~50% in KO-fed rats compared to ~40% EPA the SO-fed rats. Additionally, KO also contains high amounts of the powerful antioxidant, astaxanthin [61,62]. However, in the present study there were no significant differences in SOD, CAT or GSH-Px mRNA expression in rats fed KO compared to the other diet groups.

## Conclusions

The present study evaluated popular sources of n-3 PUFA as well as KO, a novel source of n-3 PUFAs. Higher PL content in KO compared to fish oils has lead to commercial claims of enhanced digestibility which improves n-3 PUFA tissue deposition and greater oxidative stability. Based on our results, rats fed KO had lower DHA digestibility and brain incorporation compared to the fish oil sources, SO and TO. Despite KO being rich in the antioxidant astaxanthin [26,62], lipid oxidation was not decreased and gene expression of antioxidant defense enzymes was not increased. On the other hand, rats fed SO and TO had the highest n-3 PUFAs digestibility and in turn, tissue accretion. Lipid oxidation was not increased in either SO or TO-fed rats despite higher tissue DHA deposition. On the basis that the optimal n-3 PUFA sources should provide high digestibility and efficient tissue incorporation with the least tissue lipid peroxidation, TO and SO appeared to be the sources of n-3 PUFAs most favorable to health.

## List of abbreviations

ALA: α-linolenic acid; ARA: arachidonic acid; CAT: catalase; DHA: docosahexaenoic acid; EPA: eicosapentaenoic acid; FO: flaxseed oil; GSH-Px: glutathione peroxidase; H_2_O_2 _hydrogen peroxide; KO: krill oil; LA: linoleic acid; LC-PUFA: long-chain polyunsaturated fatty acids; Cu/Zn SOD: copper/zinc superoxide dismutase; COX II: cyclooxygenase II; CVD: cardiovascular disease; MDA: malondialdehyde; Mn SOD: manganese superoxide dismutase; MO: menhaden oil; n-3 PUFAs: omega-3 polyunsaturated fatty acids; n-6 PUFAs: omega-6 polyunsaturated fatty acids; PGE_2_: prostaglandin E_2_; PL: phospholipids; RT-qPCR: real-time quantitative polymerase chain reaction; SO: salmon oil; TAC: total antioxidant capacity; TG: triglycerides; TBARS: thiobarbituric acid reactive substances; TO: tuna oil; TXB_2_: thromboxane B_2_.

## Competing interests

The authors declare that they have no competing interests.

## Authors' contributions

JCT conceived and designed the study, oversaw all aspects of the study, assisted with the data analysis and interpretation, and wrote the manuscript. SNA conducted the experiments, performed the data analysis, and assisted in the preparation of the manuscript. JCG assisted in the design of the study, contributed to the intellectual content, supervised the animal feeding experiment, and supervised the TLC and the colorimetric assays. VAB critically reviewed the manuscript for intellectual content, assisted with the experimental design, and supervised the RT-qPCR. LAC assisted with the animal experiments, fatty acid analysis, TLC, colorimetric assays, data preparation, and interpretation. All authors have read and approved the final manuscript.
